# High-throughput 3D whole-brain quantitative histopathology in rodents

**DOI:** 10.1038/srep20958

**Published:** 2016-02-15

**Authors:** Michel E. Vandenberghe, Anne-Sophie Hérard, Nicolas Souedet, Elmahdi Sadouni, Mathieu D. Santin, Dominique Briet, Denis Carré, Jocelyne Schulz, Philippe Hantraye, Pierre-Etienne Chabrier, Thomas Rooney, Thomas Debeir, Véronique Blanchard, Laurent Pradier, Marc Dhenain, Thierry Delzescaux

**Affiliations:** 1 Commissariat à l’Energie Atomique (CEA) – Molecular Imaging Research Center (MIRCen), 92265 Fontenay-Aux-Roses, France; 2 Brain and Spine Institute (ICM), 75013 Paris, France; 3 Sanofi, Therapeutic Strategy Unit Aging, 91380 Chilly-Mazarin, France; 4 IPSEN Innovation, 91140 Les Ulis, France

## Abstract

Histology is the gold standard to unveil microscopic brain structures and pathological alterations in humans and animal models of disease. However, due to tedious manual interventions, quantification of histopathological markers is classically performed on a few tissue sections, thus restricting measurements to limited portions of the brain. Recently developed 3D microscopic imaging techniques have allowed in-depth study of neuroanatomy. However, quantitative methods are still lacking for whole-brain analysis of cellular and pathological markers. Here, we propose a ready-to-use, automated, and scalable method to thoroughly quantify histopathological markers in 3D in rodent whole brains. It relies on block-face photography, serial histology and 3D-HAPi (Three Dimensional Histology Analysis Pipeline), an open source image analysis software. We illustrate our method in studies involving mouse models of Alzheimer’s disease and show that it can be broadly applied to characterize animal models of brain diseases, to evaluate therapeutic interventions, to anatomically correlate cellular and pathological markers throughout the entire brain and to validate *in vivo* imaging techniques.

Imaging pathophysiological markers in animal models is essential to understand brain disorders and to develop new therapies. To date, microscopic imaging of immunohistochemically-stained tissue section is the state-of-the art technique to reveal specific biomarkers in the brain. With the rising use of automated slide staining systems and whole slide imaging microscopes, the capabilities for production of histopathological material as well as image digitization have dramatically increased^1^. Histopathology is entering the era of big data but quantitative analysis remains a bottleneck. Adapted tools are still lacking to mine brain histopathological data and extract valuable biological information. Indeed, current standards for the analysis of brain histopathological markers heavily rely on manual intervention to delineate regions of interest (ROIs) and quantify the staining. Hence, data collection is usually restricted to a few tissue sections and a few manually defined brain ROIs. While this can be sufficient for many studies, it drastically limits the scope of the analysis in studies with low prior knowledge about biomarkers of interest, if any. It also heightens the risk of inter-subject and intra-subject variability. Moreover, as brain histological processing relies on tissue sectioning, direct spatial correlation of histopathological two-dimensional (2D) information with *in vivo* three-dimensional (3D) imaging modalities such as magnetic resonance imaging (MRI) and positron emission tomography (PET) is arduous^2^.

Several approaches have been proposed for brain *ex vivo* 3D microscopy imaging. Notably, serial electron microscopy have been used for 3D reconstruction of small tissue volumes at the nanoscopic scale^3^,^4^ and allowed the fine analysis of neuronal networks^5^,^6^,^7^,^8^. At the microscopic scale, serial optical microscopy enabled the visualization of neuronal tracers and blood vessels throughout the entire brain^9^,^10^,^11^,^12^. Recently, clearing techniques such as ultramicroscopy^13^, CLARITY^14^, SeeDB^15^, CUBIC^16^ and 3DISCO^17^, have drawn considerable attention. Clearing protocols have been proposed as a preprocessing step to optically image thick tissues in 3D. Thick tissue immunostaining is possible with some of the aforementioned techniques but it requires long incubations to allow for antibody diffusion. Thus, for practical use, the brain often needs to be cut into thick sections before immunostaining^18^,^19^.

Although, 3D reconstruction of brain histological section images has already been in use^20^,^21^,^22^,^23^,^24^, there is no generic method for quantifying cellular and pathological markers at the whole-brain level. Also, a pending question is how to make sense of this large amount of data to provide new biological insight. To address these issues, we propose an integrated approach that relies on block-face photography^25^,^26^, serial histology and 3D-HAPi (3D Histology Analysis Pipeline), an innovative image analysis pipeline freely available for non-commercial use (http://brainvisa.info). The method is scalable so that whole-brain analysis of multiple markers can be performed in dozens of animals in a short time. The proposed approach relies on two strategies in order to make sense of whole-brain histopathological data: (1) an ontology-based analysis which is used to extract information about the amount of biomarkers of interest at many anatomical levels of the brain and (2) a multimodal analysis which allows to spatially correlate several histopathological biomarkers across the whole-brain and to characterize signal from *in vivo* imaging with 3D histopathology. As a proof of concept of our method usability, we studied well-known mouse models of cerebral Aβ peptide deposition which is a neuropathological hallmark of Alzheimer’s disease^27^. In these mice, we thoroughly characterized Aβ peptide deposition, we evaluated the brain-wide effect of a new anti-amyloid immunotherapy, we spatially correlated neuroinflammatory cell markers with Aβ peptide deposition across the whole brain and we registered 3D histopathological data with *in vivo* contrast-enhanced MRI.

## Results

### An integrated method for 3D whole-brain histopathology

**Figure 1:**
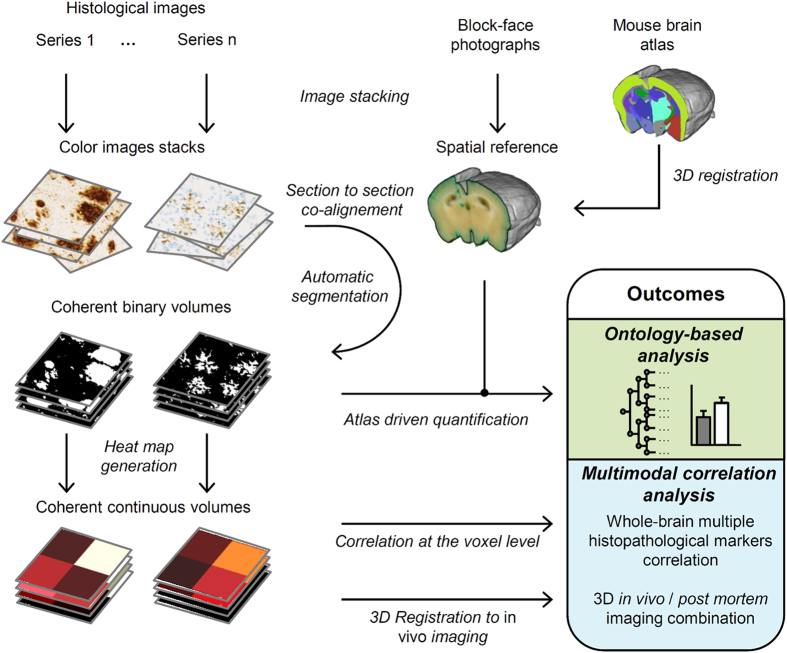
3DHAPi image analysis steps and outcomes. First, block-face photographs are stacked to provide a spatial reference (top-middle). Histology images are stacked for each series (top-left). Then each histology image is co-registered with its corresponding block-face photograph, segmented and, converted to heat map for multimodal analysis (left). An MRI-based mouse brain atlas is registered to the block-face photographic volume (top-right). The registered atlas labels and the registered segmented histology volumes are used for the ontology-based analysis.

The datasets come from studies involving two transgenic mouse strains: the APP_sl_/PS1_M146L_^28^ and the APP_swe_/PS1dE9^29^. Detailed information about mouse models and experimental procedures can be found in Supplementary Table S1. We developed a standard protocol which key principles are briefly described below and illustrated on Figure. 1.

**Figure 2:**
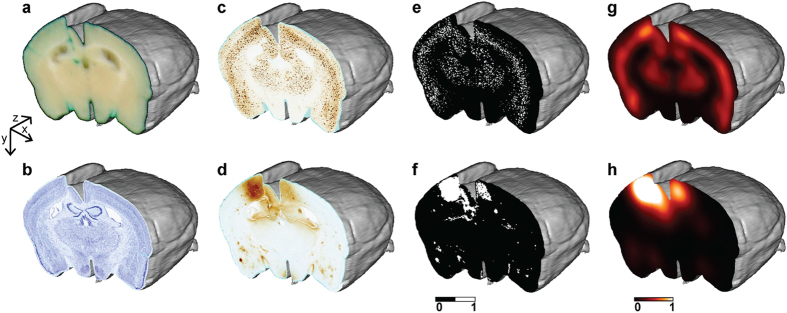
3D histopathological volumes. (**a**) Block-face photographic volume, used as a spatial reference to reconstruct histological images in 3D. (**b–h**) Reconstructed histological volumes. (**b**) Nissl volume displaying brain anatomy. (**c**) 6E10 immunohistochemistry (IHC) volume showing Aβ peptide deposits. (**d**) Anti-IgG IHC volume highlighting blood brain barrier (BBB) disruptions. (**e**) Segmented 6E10 IHC volume. (**f**) Segmented Anti-IgG IHC volume. Continuous and quantitative heat map volumes for (**g**) Aβ peptide deposits and (**h**) blood brain barrier (BBB) disruptions.

First, brains are coronally sectioned along the rostro-caudal axis from the frontal pole to the caudal end of the cerebral cortex and block-face photographs are recorded during the cutting process. Because block-face photographs are taken prior to tissue sectioning at the exact same position, the resulting image stacks yield 3D reference brain volumes for each subject that respects the 3D geometry of the brains (Fig. 2a). Serial interleaved coronal sections spanning the entire cerebrum are stained for specific markers (in multimodal experiments with 4 or 5 markers, each series typically contains about a hundred of sections). Sections are then digitized by optical bright-field imaging (whole slide imaging microscopy or flatbed scanner).

Image processing steps are all integrated into the 3D-HAPi pipeline (Supplementary Figure S1). First, images are co-aligned with their corresponding block-face photographs to generate 3D reconstructed datasets (Fig. 2b–d). 3D histology spatial resolution is defined by bright-field imaging resolution in the coronal plane and by section thickness and section sampling in the rostro-caudal axis. Each staining is then automatically segmented on histological images with a machine learning classifier^30^ providing binary volumes ready to be quantified (Fig. 2e,f). Segmentation performances are validated by visual inspection and by calculating F1 scores (Supplementary Table 2). To automatically parcellate the brain into anatomical regions, an MRI-based brain atlas is registered to the block-face photographic volume by successively estimating and applying rigid, affine and non-linear transformations using a methodology developed and validated by Lebenberg *et al.*^31^. As histological volumes are all registered to the block-face photographic volume, precise registration of the MRI-based brain atlas with the block-face photographic volume ensures that histological volumes are correctly parcellated (Supplementary Fig. S2). In addition, we adapted the NeuroNames brain ontology^32^ to compute results at different anatomical levels (Supplementary Fig. S3). Therefore, the marker load can be quantified in several dozens of hierarchically organized ROIs.

High-resolution binary volumes can be summarized in lower-resolution continuous and quantitative heat map volumes, which require less computer memory and computation time while preserving local quantitative information (Fig. 2g,h). In multimodal experiments, heat map volumes can be used to spatially correlate several histopathological biomarkers and to correlate histopathological quantitative data with *in vivo* imaging.

In our experimental settings, it took about 4 days to process a whole-mouse brain with 2 histopathological markers: 2 days for brain sectioning, block-face photography imaging and subsequent staining (2 series of 114 sections), 1 day for image digitization (lateral resolution: 5 μm) and 1 day for image processing on an up-to-date personal computer (2.83 GHz processor, 16 GB RAM). If computer resources are sufficient, several brain datasets can be analyzed in parallel and submicroscopic resolution images can be processed.

### 3D versus 2D histopathology comparison

**Figure 3:**
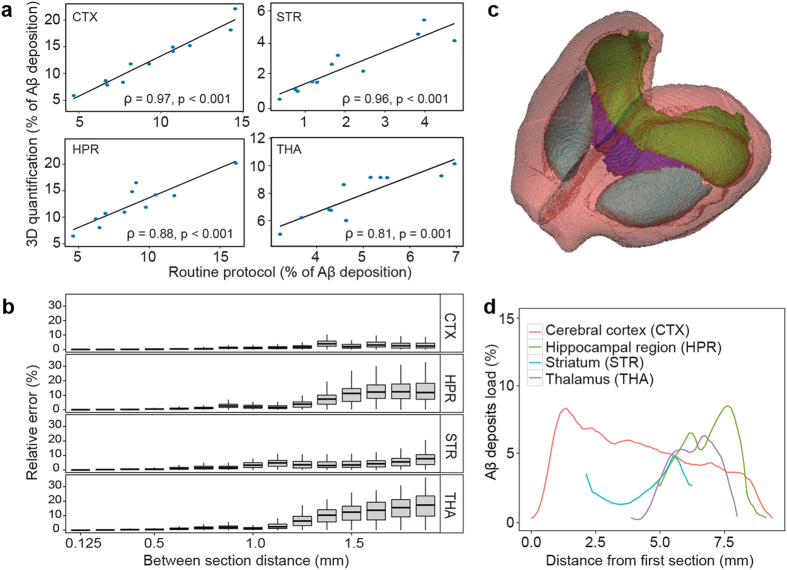
3D quantification and 2D quantification comparison. (**a**) Correlations of Aβ deposition quantifications using brain-wide histopathology and a routine quantification protocol in the 4 selected ROIs in APP/PS1 mice (N = 11, Spearman’s rank correlation). A linear regression line is shown in black. (**b**) Relative error of Aβ deposition measures when increasing the distance between analyzed equidistant sections in APP/PS1 mice (N = 11). Boxplots upper and lower hinges correspond to the first and third quartiles. The whiskers extend from the hinges to the highest or lowest values that are within 1.5 × inter-quartile-range. Bold lines represent median values. See Methods section for relative error definition. (**c**) A 3D rendering of 4 selected ROIs (cerebral cortex in red, striatum in light blue, hippocampal region in green and thalamus in purple). (**d**) Aβ deposition distribution profile along the rostro-caudal axis in an APP/PS1-13C3a mouse in the 4 selected ROIs.

First, we sought to compare Aβ load quantification between our 3D whole-brain quantification and an independently performed 2D routine quantification protocol with a few sections per ROI. We correlated results for both methods in 4 ROIs (cerebral cortex, striatum, hippocampal region and thalamus) in 11 APP/PS1 transgenic mice (Fig. 3a). Overall, the two methods well correlated but showed various levels of agreement according to the ROI (Spearman’s *ρ* = 0.97, p < 0.001 in the cerebral cortex; *ρ* = 0.96, p = 0.001 in the striatum; *ρ* = 0.88, p = 0.001 in the hippocampal region; *ρ* = 0.81, p = 0.001 in the thalamus). We further attempted to explain differences between ROIs, by investigating the effect of lowering section sampling on Aβ load quantification. Using 3D histopathological data, simulations were performed for each animal by progressively discarding equidistant sections from the analysis and generating all possible combinations of sections at a given sampling rate. We then quantified Aβ deposition for each subset and calculated the relative extent to which the subset quantification deviated from reference 3D quantification. Figure 3b shows the effect of section sampling lowering on Aβ deposition quantification relative error. When decreasing sampling rate, the relative error remained relatively low in the cerebral cortex and in the striatum but increased in the thalamus and the hippocampal region, up to median relative errors of, respectively, 17.3% and 11.9% for subsets of 1.875 mm-separated sections. This corresponds to subsets of 2 or 3 sections per ROI which is fairly common in the literature (a literature survey on commonly used techniques for Aβ deposition quantification is presented in Supplementary Figure S4). To better understand discrepancies between ROIs, Aβ load rostro-caudal dispersion was quantified for each ROI (coefficients of variation: 39% in the cerebral cortex, 37% in the striatum, 44% in the hippocampal region and 55% in the thalamus, Fig. 3c,d). This indicates that differences between the 2D quantification approach and 3D histopathology can be explained by variations in biomarker spatial distribution inside ROIs.

### Ontology-based mouse model characterization

**Figure 4:**
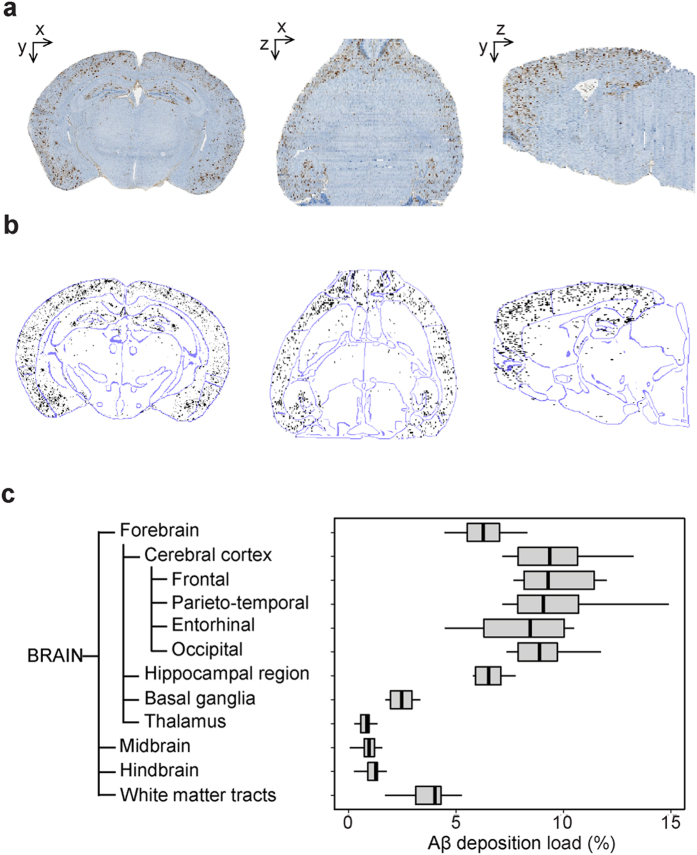
Aβ deposition in APP/PS1dE9 transgenic mice. (**a**) A representative reconstructed Aβ deposits volume in the coronal (left), axial (middle) and sagittal (right) views. (**b**) Segmented Aβ deposits (black) superimposed with the atlas contours (blue) computed with a Deriche filter. (**c**) Aβ deposition quantification in 13.5-month-old APP/PS1dE9 mice (N = 7). Boxplot representation is the same as in Fig. 3b.

One possible application of our method is to thoroughly characterize animal models. After reconstruction of 7 brains from 13.5 months old APP/PS1dE9 mice (Fig. 4a), we took advantage of the brain ontology to quantify Aβ peptide deposition at different levels of the brain (Fig. 4b,c). Aβ deposition, as expressed as a percentage of volume occupied by Aβ deposits in a given ROI, peaked in the cortical regions and the hippocampus. Those results agree with previously published works reporting massive amyloid deposition in these regions in aged APP/PS1dE9 mice^29^. Furthermore, ontology driven analysis highlighted infrequently examined ROIs presenting substantial Aβ deposition such as basal ganglia and white matter tracts.

### Therapeutic intervention evaluation

**Figure 5:**
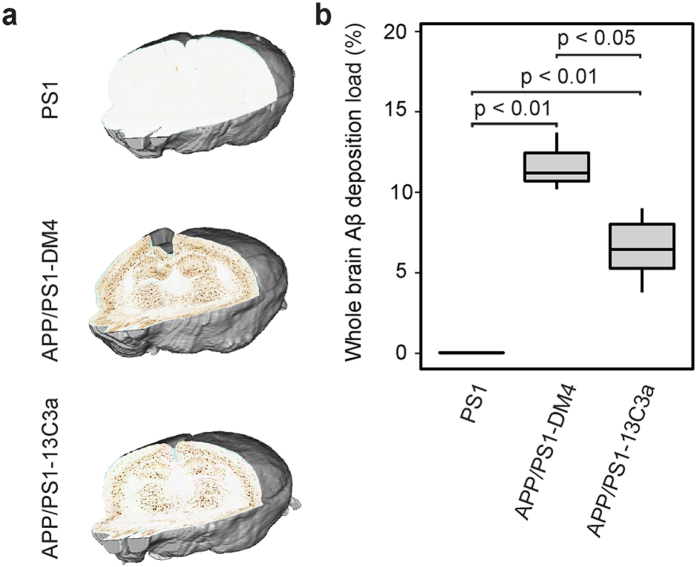
Brain-wide anti-amyloid effect of 13C3a. (**a**) Reconstructed Aβ deposition IHC volumes for one representative brain per group. (**b**) Reduction of Aβ deposition in APP/PS1 mice treated with 13C3a (n = 8) compared to mice treated with DM4 (n = 3). PS1 mice are shown as negative controls (n = 4) (Mann-Whitney tests, boxplot representation is the same as in Fig. 3b).

Preclinical drug evaluation is another major application. To illustrate this, we evaluated the effect of a new anti-amyloid immunotherapy in 8-months-old APP/PS1 transgenic mice in the entire brain and in four ROIs (cerebral cortex, striatum, hippocampal region and thalamus). In addition, Aβ deposits free PS1 mice were included as negative controls (Fig. 5a). As expected, Aβ deposition load was close to none in brains of PS1 mice (Fig. 5b). We compared Aβ deposition between APP/PS1 mice treated with either the anti-amyloid immunotherapy (13C3a) or a control antibody (DM4). In brains of APP/PS1 mice, 13C3a lowered Aβ deposition compared with DM4 (Mann-Whitney test, p < 0.05; Fig. 5a,b). Besides, we detected a statistically significant Aβ lowering effect of 13C3a in the cerebral cortex, the striatum and the thalamus but not in the hippocampal region (Supplementary Table S3). Nevertheless, results regarding the effect of 13C3a should be taken carefully due to the small sample size of this study.

### Multimodal exploration of the brain using heat maps

**Figure 6:**
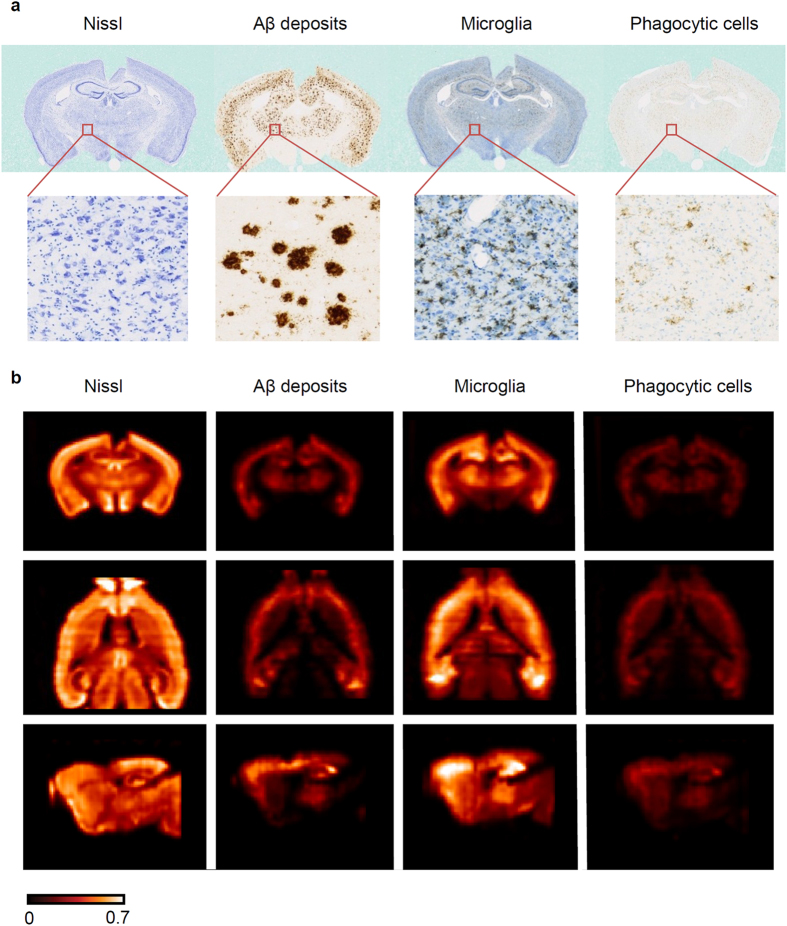
Multiple marker 3D histopathology. (**a**) Histological coronal section images at a lateral resolution of 0.44 μm. Left: Nissl staining in blue; middle-left: Aβ deposits in brown (6E10 IHC); middle-right: microglia in black (Iba-1 IHC) and Nissl counterstaining in blue; right: phagocytic cells in brown (CD68 IHC) and light Nissl counterstaining in blue. (**b**) Pathological and cell markers heat maps with 150520 voxels each. Top: coronal view; middle: axial view; bottom: sagittal view.

**Figure 7:**
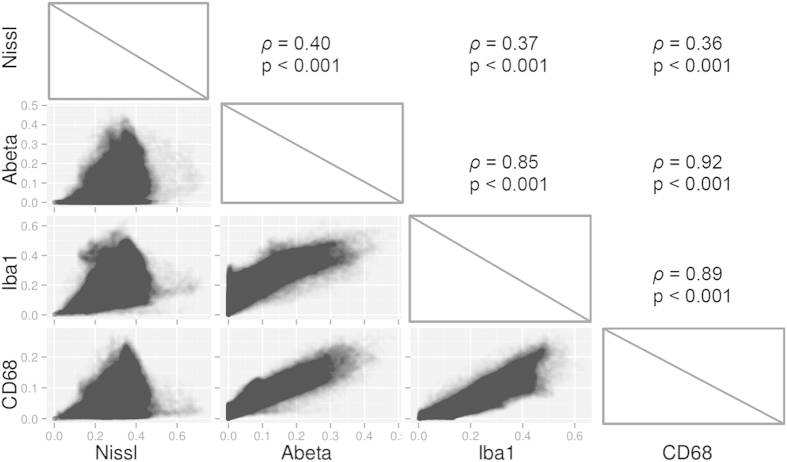
Multiple marker spatial correlation. Spearman’s rank correlations between markers are shown in the upper panels while corresponding scatter plots are shown in the lower panels. Scatter plots data points have 10% opacity to allow for better visualization.

We studied the coupling between Aβ deposition and several cellular markers in one 8-months-old APP/PS1-DM4 mouse. Several markers were quantified at microscopic resolution (lateral resolution: 0.44 μm) in this mouse: Aβ peptide deposits (6E10 positive), phagocytic cells (CD68 positive), microglial cells (Iba1 positive), and Nissl-bodies (Fig. 6a). Brain-wide quantitative heat maps with an isotropic resolution of 125 μm^3^ were derived from high-resolution segmented images for each marker (Fig. 6b). A total of ~150000 voxels spanning the entire brain were extracted for each marker. As heat map volumes were all in the same spatial referential, we correlated voxel values between markers in order to assess their degree of spatial dependence (Fig. 7). Interestingly, among all between-marker correlations, Aβ deposits and phagocytic cells showed the strongest association (Spearman’s *ρ* = 0.92, p < 0.001). This demonstrates that Aβ deposition is tightly and widely associated with cells presenting a phagocytic phenotype. Nissl staining only weakly correlated with the remaining markers (Spearman’s *ρ* = 0.40, p < 0.001 versus Aβ deposition, Spearman’s *ρ* = 0.37, p < 0.001 versus microglial cells, Spearman’s *ρ* = 0.36, p < 0.001 versus phagocytic cells). Microglial cells strongly correlated with both Aβ deposition (Spearman’s *ρ* = 0.85, p < 0.001) and phagocytic cells (Spearman’s *ρ* = 0.89, p < 0.001) which is consistent with the fact that microglial cells are involved in Aβ plaque phagocytosis^33^.

### *In vivo*-*ex vivo* imaging signal concordance

**Figure 8:**
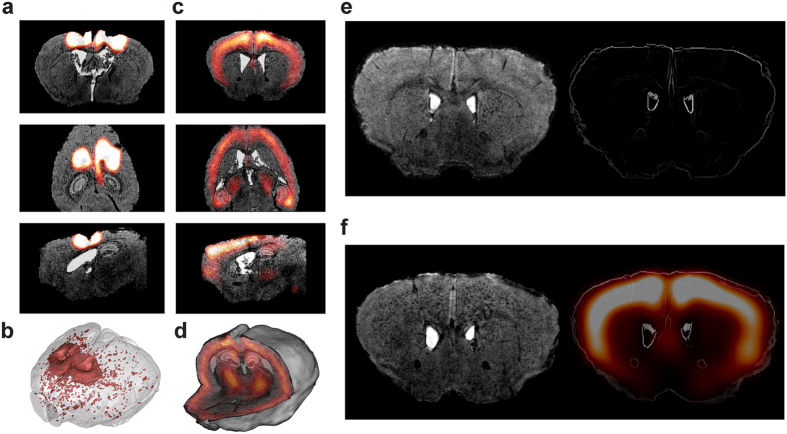
*In vivo* – *ex vivo* imaging confrontation. (**a**) BBB disruption heat map in coronal (top), axial (middle) and sagittal (bottom) views. (**b**) 3D rendering of BBB disruptions detected by 3D-HAPi in one APP/PS1-DM4 mouse presenting this particular phenotype. (**c**) Aβ deposition heat map in coronal (top), axial (middle) and sagittal (bottom) views. (**d**) 3D rendering of Aβ deposition heat map with *in vivo* MRI. (**e**) A PS1 mouse that does not display Aβ deposition neither on *in vivo* MRI (left) nor on Aβ deposition heat map (right) and (**f**) an APP/PS1 mouse displaying hypo-intense spots in the cerebral cortex (left) with a similar distribution to the Aβ deposition heat map (right). Aβ deposition heat maps have been superimposed with *in vivo* MRI contours to allow better visualization.

In APP/PS1 and PS1 mice, local blood brain barrier disruptions (BBB) were induced by bilateral intracerebroventricular injection of a contrast agent prior to *in vivo* MRI. After euthanasia, serial anti-IgG immunohistochemistry (IHC) was performed to reveal areas of antibody leakage from blood to brain parenchyma. Brain-wide heat maps from APP/PS1 and PS1 mice were warped on their corresponding contrast-enhanced *in vivo* MRI using the block-face photography reference volume as an intermediate between heat maps and MRI. Registration quality was visually validated by superimposing contours of registered block-face photographic volumes on *in vivo* MRI volumes (Supplementary Figure S5). Massive IgG leakage was observe on anti-IgG immunochemistry-derived heat maps and collocated with injection sites spotted on MRI (Fig. 8a). In addition, one APP/PS1-DM4 mouse was found with brain-wide BBB disruptions (Fig. 8b and Supplementary Movie S1) which were not seen on MRI. Finally, Aβ deposition heat maps were warped on MRI (Fig. 8c–f and Supplementary Movie S2). No Aβ deposition was detected in MRI and in Aβ deposition heat maps of PS1 mice (Fig. 8e). Interestingly, hypo-intense spots were specifically observed on MRI in the cerebral cortex of APP/PS1 mice with a distribution corresponding to that of Aβ deposition heat maps (Fig. 8f) which confirms previous studies showing that Aβ aggregates can be detected by contrast-enhanced MRI^34^,^35^.

## Discussion

Although previous techniques have been proposed for high-resolution 3D imaging of the brain the proposed method constitutes, to our knowledge, a unique start-to-end exploratory approach for whole-brain histopathological imaging analysis. Indeed, it performs rapid and automated quantitative analysis which allows for large scale animal studies in time-constrained environments. In the present work, we have illustrated this method on widely studied mouse models of Aβ peptide deposition to show its usability for ontology-based biomarker quantification, 3D registration to *in vivo* imaging and brain-wide correlations between biomarkers. As 3D-HAPi is part of the open-source BrainVISA neuroimaging software, its field of applications can be extended by custom-developed modules. Currently, the first version of 3D-HAPi comes with a single classification algorithm dedicated to the segmentation of neuropathological markers but new classification algorithms could be easily plugged into the pipeline to segment more challenging objects such as neuronal processes.

Reliably measuring the amount of a marker of interest in various anatomical regions is primordial to evaluate the effect of experimental interventions. Here, regional Aβ load measures could be performed thanks to unsupervised atlas registration. As a consequence, successful biomarker quantification strongly relies on accurate atlas registration. Image registration is based on numerical optimization and intrinsically involves some level of approximation which is important to evaluate. Consequently, as 3D histopathology allows the quantification of very tiny objects in the brain such as Aβ plaques, it is possible that despite good overall registration, objects located close to anatomical borders are incorrectly attributed to a region owing to small registration errors which extent is nonetheless greater than the size of the objects at study. The present work does not include a quantitative validation of the registration results but the employed methodology have been extensively validated by Lebenberg *et al.*^31^ for multimodal registration between MRI-based atlas and block-face photography. Results from Lebenberg *et al.*^31^ indicated that registration is very accurate for relatively large regions but can be less accurate for small regions of the brain because the estimated transformations are not precise enough to match small regions without much contrasts. This is why, in this study, we sought to design a brain ontology which allows to merge small regions into larger regions where the measures can be performed with high accuracy. Besides, another advantage of the ontology is to enable the selection of the most relevant, possibly overlapping, regions for each particular study whereas an atlas has a limited number of non-overlapping regions. While available brain atlases include different regions of interest, our brain ontology has been designed according to the NeuroNames consensus neuroanatomical ontology^32^, therefore providing a unified anatomical framework for biomarker quantification allowing for harmonization between studies.

In this work, ontology-based analysis allowed to highlight new anatomical regions of Aβ deposition in APP/PS1dE9 and to detect the brain-wide Aβ load lowering effect of the 13C3a immunotherapy. Thus, 3D ontology-based analysis can be broadly used to thoroughly phenotype animal models and evaluate experimental interventions such as new therapies. Importantly, this work demonstrates that 3D ontology-based analysis can provide significant improvement of quantification accuracy compared to more classical approaches which are based on the analysis of a few sections in 2D. Besides, it avoids variability due to operator-dependent delineation of ROIs, thus allowing for more reproducible results.

Nonetheless, compared to conventional 2D protocols, 3D whole-brain quantification comes at a cost in immunohistochemistry reagent consumption as well as in tissue processing and image acquisition times. Therefore, in essence, our approach is best adapted for exploratory studies, when *a priori* knowledge about biomarker spatial distribution is low or when studying experimental perturbations with unknown localizations. In those cases, 3D histopathology can detect local effects or subtle changes between groups that could have been missed by a more conventional approach. For instance, it is believed that pathological aggregates such as Aβ plaques spread to distant brain regions through functional networks but full characterization of the regions that are affected by pathology and the relationship between pathology and functional connectivity would benefit from 3D quantification.

Besides ontology-based approach, we propose to use histopathology derived heat maps which are suitable to integrate quantitative information from tremendous amount of microscopic data. In this work, heat maps enabled to assess the brain-wide spatial relationships between Aβ deposition and 3 cellular markers. Notably, we found a very strong linear relationship between Aβ load and CD-68 load which indicates that Aβ deposition is tightly associated with phagocytic cells and that this association is stationary throughout the brain.

Heat maps can also be used to efficiently warp 3D quantitative histological data with 3D *in vivo* imaging modalities. This is of primary importance to validate new imaging instrumental devices, MR sequences, MR contrast agents and PET radiotracers. Here, we visually evaluated the concordance between *in vivo* contrast-enhanced MRI and *ex vivo* histology-derived Aβ deposition heat maps. Segmentation algorithms are under development in order to quantify Aβ deposits on *in vivo* MRI scans. This will allow to quantitatively validate *in vivo* measurements of Aβ deposition using 3D quantitative histopathology.

An exciting perspective of this work is to take advantage of whole-brain heat maps for voxel-wise studies. In neuroscience clinical research, voxel-wise statistical analysis is commonly adopted to unveil local changes between sets of functional PET or MRI quantitative images^36^. This technique has been successfully translated for small animal imaging^37^. More recently, voxel-wise analysis of *post mortem* 3D reconstructed autoradiography data enabled high-resolution exploratory functional studies^38^,^39^,^40^. Applying this approach to analyze 3D microscopic data is however challenging because histopathological images are not quantitative and their resolution is very high. Histopathology-derived heat maps alleviate this issues which paves the way for voxel-wise analysis of cellular and pathological markers in rodents. This could complement the atlas-based analysis in order to detect local changes between groups of animals without any regional *a priori*.

Another perspective is to extend our approach to cellular object-level analysis. Currently, 3D-HAPi analyzes biomarkers in term of marker load. This occults object-level information such as cell counting, size and shape. Multiple cellular features could be computed at the level of the whole-brain and summarized in the form of multivariate heat maps. This could provide the opportunity to get deeper insight into neuroanatomy and to improve our knowledge of cellular mechanisms involved in disease states. Finally, as immunostaining thick samples have been made possible thanks to advances in tissue clarification, our method could be extended to reconstruct thick immunostained brain tissue images into whole-brain volumes and analyze brain cell structures in 3D throughout the brain. Nonetheless, one remaining major challenge is to enable robust cell individualization in regions where cellular density is particularly high. Developments in high-performance computing are also ongoing to enable whole-brain analysis in reasonable time despite the tremendous amount of data to be processed.

## Methods

### Animal models and *in vivo* MRI

All animal experiments were done according to the European recommendations (86/609/EEC) and conformed to the ethical guidelines of the French National Charter on the Ethic of Animal Experimentation. All experiments were approved by the “Direction Départementale des Services Vétérinaires de l’Essonne” (authorization number 91–326).

In the first study, ten male APP_swe_/PS1dE9 mice were initially included in the animal model characterization study. Three mice died prematurely. Mice were euthanized at the age 13.5 months with lethal injection of sodium pentobarbital; brains were rapidly removed and snap-frozen in isopentane. Seven brains were kept for further analysis.

In the second study, twenty female APP_sl_/PS1_M146L_ mice were initially included at the age of 5 months in the therapy evaluation study. They received weekly intraperitoneal injections of either 13C3a, an anti-amyloid antibody^41^ (Sanofi, 10 mg/kg) or DM4, a control antibody (Sanofi, 10 mg/kg) for 3 months. In addition, 4 PS1_M146L_ mice were included in the study as negative controls (PS1_M146L_ mice are amyloid plaque free). At 8 months of age, APP_sl_/PS1_M146L_ mice as well as PS1_M146L_ mice received intracerebroventricular injections of gadolinium and 3D Gradient-echo MRI scans (*x*, *y*, *z* resolution: 29 × 29 × 117μm^3^) were recorded on a 7T-Spectrometer (Agilent) prior to euthanasia and brain removal. This protocol was used to visualize Aβ deposits *in vivo*^35^. At this stage, due to the high mortality rates of APP_sl_/PS1_M146L_ transgenic mice, especially in the control-treated group, eight 13C3a-treated APP_sl_/PS1_M146L_ mice and three DM4-treated APP_sl_/PS1_M146L_ mice were included for analysis. All the APP_sl_/PS1_M146L_ mice were also included for the comparison between 3D analysis and routine 2D quantification. Summarized information on both studies is provided in Supplementary Table S1.

### Histology

Brains from APP_swe_/PS1dE9 mice were processed as described in details in Supplementary Protocol S1. Briefly, brains were cut into 20-μm-thick serial coronal brain sections from the frontal pole to the caudal end of the cerebral cortex. Block-face photographs were taken for every fourth section (Powershot G5 Pro, Canon) at a lateral resolution of 27 μm. One series was used for Nissl-staining and one for Aβ peptide deposits immunohistochemistry (IHC) (BAM10 monoclonal antibody, Sigma-Aldrich). To perform reproducible staining across the whole study, we used the Ventana Discovery XT slide staining system (Ventana Medical Systems, Inc.).

Brains from APP_sl_/PS1_M146L_ and PS1_M146L_ mice were processed by NeuroScience Associates. Formalin-fixed brains were all embedded in one green-colored solid matrix to obtain a sharp contrast between the embedding material and the cerebral tissue. Brains were subsequently frozen and cut into 25 μm-thick serial coronal brain sections from the frontal pole to the caudal end of the cerebral cortex. Block-face photographs were recorded at a lateral resolution of 33 μm every fifth section (EOS Rebel T2i, Canon). The first series of sections was used for Nissl staining, the second for Aβ deposits staining (6E10 monoclonal antibody IHC), the third for phagocytic cells staining (anti-CD68 monoclonal antibody IHC) with a Nissl counterstaining, the fourth for blood brain barrier (BBB) disruptions staining (anti-IgG monoclonal antibody IHC) the fifth for microglia staining (anti-Iba1 monoclonal antibody IHC) with a Nissl counterstaining.

### Histology digitization

A flatbed scanner (ImageScanner III, G.E. Healthcare) was used to digitize all Nissl-stained series of sections (lateral resolution: 21 μm) and all series of sections stained for Aβ deposits (lateral resolution: 5 μm). For all APP_sl_/PS1_M146L_ mice, a subset of 8 Aβ-deposit-stained sections per animal was acquired at a lateral resolution of 0.35 μm with an Olympus VS120-S5 whole slide imaging microscope. These latter images were used to perform the routine 2D quantification protocol of Aβ deposition. Furthermore, for one APP_sl_/PS1_M146L_ -DM4 mouse, the Aβ deposits series and the cellular staining series (Nissl, Iba-1 IHC for microglia and CD-68 IHC for phagocytic cells) were digitized with a Zeiss Axio Scan.Z1 whole slide imaging microscope at a lateral resolution of 0.44 μm. This high-resolution microscopy dataset was used to investigate brain-wide spatial correlation between markers.

### Routine 2D quantification protocol for Aβ deposition in APP/PS1 mice

A 2D quantification protocol was performed in order to compare current analysis practices with 3D histopathology. Image analysis was performed with Mercator software (Explora Nova) by an expert blinded to the quantification results obtained with the 3D quantification approach. Four ROIs (cerebral cortex, hippocampal region, striatum and thalamus) were selected for the analysis. The cerebral cortex was manually outlined on 8 sections per animal. The hippocampal region, the striatum and the thalamus were present and were outlined on 3 out of the 8 selected sections per animal. A threshold that appropriately separated Aβ peptide deposits from the remaining unstained tissue was manually defined on one section and then applied to all images. Finally, Aβ deposition load was calculated in each selected ROI of each animal.

### 3D reconstruction

All the image processing steps were performed using 3D-HAPi which is part of the BrainRAT toolbox of BrainVISA, a neuroimaging software. First, block-face photographs and histological images were stacked. As block-face images were taken prior to sectioning at the same position section after section, block-face photographic volumes naturally respected the original shape of the frozen brains and were used as a 3D reference. Block-face photographic volumes were segmented with an automatic threshold operation^25^ and masked to remove background. Affine registration between histological images and corresponding block-face photographs were estimated by maximizing the correlation coefficient between images using the Block-Matching method^20^,^42^ at three resolution levels to ensure robust registration. Registration allowed to correct for deformations due to histological procedures and provided coherent histological volumes. Thus, for each animal, the block-face photographic volume and all corresponding histological volumes were reconstructed in the same spatial referential.

### 3D multimodal registration

3D registration was performed for two purposes: (1) to match an MRI-based atlas of the mouse brain (http://www.mouseimaging.ca/technologies/C57Bl6j_mouse_atlas.html)^43^ with 3D histopathological volumes and (2) to match, for every APP/PS1 mouse and PS1 mouse, 3D histopathological volumes with their corresponding *in vivo* contrast-enhanced MRI. In both cases, transformations were estimated between anatomical modalities (block-face photographic volume and (1) the atlas MRI or (2) the *in vivo* MRI) as follows. A global rigid transformation (rotation, translation) was estimated by maximizing the mutual information similarity criterion with the Powell optimizer^44^. Then, an affine registration initialized with the rigid transformation was performed by maximizing the correlation coefficient with the Block-Matching technique^20^,^42^. To improve registration locally, we performed an elastic transformation, based on free-form deformation^45^ using mutual information as the optimization criterion and the BFGS optimizer and a grid of 10 × 10 × 10 regularly spaced control points^31^. After transformations were calculated between anatomical modalities, they were either applied (1) to the atlas labels to match 3D histological volumes or (2) to 3D histological data to match *in vivo* MRI.

### Histopathological markers segmentation

Staining for markers of interest were segmented on each reconstructed histological volumes with our implementation of BioVision, a supervised machine learning algorithm^30^. This algorithm consists of a learning step to generate a model from ground-truth data and a classification step which enables to segment new images. A ground-truth data set was constructed for each biomarker by extracting small patches from high-resolution images and by manually delineating voxels belonging to 3 classes: positively stained tissue, non-stained tissue and background. In the learning step, BioVision extracts color and local intensity which are used as predictors for classification. Local intensity is particularly helpful to account for noise in neuropathology images and is calculated for each voxel as the mean of red, green and blue intensities within 4-connected voxels in the coronal plane. BioVision models the expert-based segmentation by estimating, for each class, the predictors’ joint probability distribution with a multivariate Gaussian Mixture Model. Parameters for fitting the Gaussian Mixture Model were chosen according to Chubb *et al.*^30^. In the classification step, each voxel is assigned to a class using Bayes rule. Segmentation results were assessed visually and by calculating F1 scores for biomarkers of interest as:


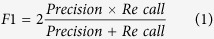


where 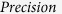
is the proportion of true positives among the voxels that are classified as positive and 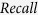 is the proportion of voxels that are classified as positives among the voxels that are positive in the ground-truth dataset.

### Ontology-driven analysis

For each animal, a MRI-based mouse brain atlas was registered on the block-face photographic volume following the protocol described above. The amount of staining (percentage of volume occupied by the staining) was computed in each of the atlas ROIs. A brain hierarchy adapted from NeuroNames^32^ was used to compute results at different anatomical levels of the brain.

### Heat map generation

In addition, quantitative and continuous heat map volumes were generated by aggregating voxels of the high-resolution segmented binary images according to *x* and *y* directions into lower resolution voxels (125 μm^3^ isotropic resolution) and attributing to these latter the value of the staining occupation ratio computed in the high-resolution images. A Gaussian smoothing with a kernel size equal to the heat map volume voxel size was applied in *x*, *y* and *z* directions.

### *In vivo*-*ex vivo* imaging confrontation

Histology heat maps were warped to *in vivo* MRI geometry in order to preserve MRI scans in their native form. This is of importance because Aβ deposits appear as hypo-intense spots on contrast-enhanced MRI^35^ and resampling operation could alter Aβ deposits geometry and hinder visual interpretation. On the contrary, histology heat maps are continuous, quantitative and can be linearly resampled without loss of information. 3D registrations were performed as described above.

### Statistics

Statistical analysis was performed using R statistical environment. For the evaluation of the effect of 13C3a immunotherapy, group comparisons were limited to 4 brain ROIs to avoid type I errors due to multiple comparisons and Mann-Whitney tests were carried out between groups. Relative error from 3D quantification was calculated as:


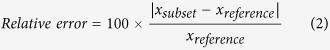


where 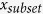 is the Aβ deposition load calculated with a simulated subset of sections and 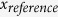 is the Aβ deposition load when all sections are taken into account. Rostro-caudal dispersion of Aβ deposition was assessed by computing coefficients of variation (CV) of single section Aβ deposition loads for each ROI and for each mouse, and then a mean CV was computed for each ROI. CV was chosen in order to provide comparable estimates of variance across mice and ROIs with various levels of Aβ deposition. Agreement between 3D histopathology quantification and the 2D routine protocol quantification as well as brain-wide spatial correlations between histopathological markers were assessed using Spearman’s rank *ρ* correlation. Non-parametric tests were chosen when data could not be assumed to follow the Gaussian distribution. Whenever possible, sample size was chosen to provide adequate statistical power.

## Additional Information

**How to cite this article**: Vandenberghe, M. E. *et al.* High-throughput 3D whole-brain quantitative histopathology in rodents. *Sci. Rep.* **6**, 20958; doi: 10.1038/srep20958 (2016).

## Supplementary Material

Supplementary Movie S1

Supplementary Movie S2

Supplementary Information

## References

[b1] Ghaznavi F., Evans A., Madabhushi A. & Feldman M. Digital Imaging in Pathology: Whole-Slide Imaging and Beyond. Annu. Rev. Pathol. 8, 331–359, doi: 10.1146/annurev-pathol-011811-120902 (2013). 23157334

[b2] Dubois A., Dauguet J. & Delzescaux T. *Ex vivo* and *in vitro* cross calibration methods, Small Animal Imaging, Springer, New York, 317–346, doi: 10.1007/978-3-642-12945-2_23 (2011).

[b3] Denk W. & Horstmann H. Serial block-face scanning electron microscopy to reconstruct three-dimensional tissue nanostructure. PLoS Biol. 2, 1900–1909, doi: 10.1371/journal.pbio.0020329 (2004). PMC524270 15514700

[b4] Knott G., Marchman H., Wall D. & Lich B. Serial section scanning electron microscopy of adult brain tissue using focused ion beam milling. J. Neurosci. 28, 2959–2964, doi: 10.1523/jneurosci.3189-07.2008 (2008). 18353998 PMC6670719

[b5] Bock D. D. *et al.* Network anatomy and *in vivo* physiology of visual cortical neurons. Nature 471, 177–182, doi: 10.1038/nature09802 (2011). 21390124 PMC3095821

[b6] Helmstaedter M. *et al.* Connectomic reconstruction of the inner plexiform layer in the mouse retina. Nature 500, 168–174, doi: 10.1038/nature12346 (2013). 23925239

[b7] Beyer J. *et al.* ConnectomeExplorer: Query-Guided Visual Analysis of Large Volumetric Neuroscience Data. IEEE Trans. Vis. Comput. Graph. 19, 2868–2877 (2013). 24051854 10.1109/TVCG.2013.142 PMC4296725

[b8] Hayworth K. J. *et al.* Imaging ATUM ultrathin section libraries with WaferMapper: a multi-scale approach to EM reconstruction of neural circuits. Front. Neural Circuits 8, 68, doi: 10.3389/fncir.2014.00068 (2014). 25018701 PMC4073626

[b9] Ragan T. *et al.* Serial two-photon tomography for automated *ex vivo* mouse brain imaging. Nat. Methods 9, 255–258, doi: 10.1038/nmeth.1854 (2012). 22245809 PMC3297424

[b10] Zingg B. *et al.* Neural Networks of the Mouse Neocortex. Cell 156, 1096–1111, doi: 10.1016/j.cell.2014.02.023 (2014). 24581503 PMC4169118

[b11] Oh S. W. *et al.* A mesoscale connectome of the mouse brain. Nature 508, 207–214, doi: 10.1038/nature13186 (2014). 24695228 PMC5102064

[b12] Wu J. *et al.* 3D BrainCV: Simultaneous visualization and analysis of cells and capillaries in a whole mouse brain with one-micron voxel resolution. Neuroimage 87, 199–208, doi: 10.1016/j.neuroimage.2013.10.036 (2014). 24185025

[b13] Dodt H.-U. *et al.* Ultramicroscopy: three-dimensional visualization of neuronal networks in the whole mouse brain. Nat. Methods 4, 331–336, doi: 10.1038/nmeth1036 (2007). 17384643

[b14] Chung K. *et al.* Structural and molecular interrogation of intact biological systems. Nature 497, 332–337, doi: 10.1038/nature12107 (2013). 23575631 PMC4092167

[b15] Ke M. T., Fujimoto S. & Imai T. SeeDB: a simple and morphology-preserving optical clearing agent for neuronal circuit reconstruction. Nat. Neurosci. 16, 1154–1161, doi: 10.1038/nn.3447 (2013). 23792946

[b16] Susaki E. A. *et al.* Whole-Brain Imaging with Single-Cell Resolution Using Chemical Cocktails and Computational Analysis. Cell 157, 726–739, doi: 10.1016/j.cell.2014.03.042 (2014). 24746791

[b17] Ertuerk A. *et al.* Three-dimensional imaging of solvent-cleared organs using 3DISCO. Nat. Protoc. 7, 1983–1995, doi: 10.1038/nprot.2012.119 (2012). 23060243

[b18] Yang B. *et al.* Single-Cell Phenotyping within Transparent Intact Tissue through Whole-Body Clearing. Cell 158, 945–958, doi: 10.1016/j.cell.2014.07.017 (2014). 25088144 PMC4153367

[b19] Tomer R., Ye L., Hsueh B. & Deisseroth K. Advanced CLARITY for rapid and high-resolution imaging of intact tissues. Nat. Protoc. 9, 1682–1697, doi: 10.1038/nprot.2014.123 (2014). 24945384 PMC4096681

[b20] Ourselin S., Roche A., Subsol G., Pennec X. & Ayache N. Reconstructing a 3D structure from serial histological sections. Image Vis. Comput. 19, 25–31, doi: 10.1016/s0262-8856(00)00052-4 (2001).

[b21] Dauguet J. *et al.* Three-dimensional reconstruction of stained histological slices and 3D non-linear registration with *in-vivo* MRI for whole baboon brain. J. Neurosci. Methods 164, 191–204, doi: 10.1016/j.jneumeth.2007.04.017 (2007). 17560659

[b22] Amunts K. *et al.* BigBrain: An Ultrahigh-Resolution 3D Human Brain Model. Science 340, 1472–1475, doi: 10.1126/science.1235381 (2013). 23788795

[b23] Hebert F. *et al.* Cortical atrophy and hypoperfusion in a transgenic mouse model of Alzheimer’s disease. Neurobiol. Aging 34, 1644–1652, doi: 10.1016/j.neurobiolaging.2012.11.022 (2013). 23273599

[b24] Grand’Maison M. *et al.* Early cortical thickness changes predict beta-amyloid deposition in a mouse model of Alzheimer’s disease. Neurobiol. Dis. 54, 59–67, doi: 10.1016/j.nbd.2013.02.005 (2013). 23454197

[b25] Dubois A. *et al.* Detection by voxel-wise statistical analysis of significant changes in regional cerebral glucose uptake in an APP/PS1 transgenic mouse model of Alzheimer’s disease. Neuroimage 51, 586–598, doi: 10.1016/j.neuroimage.2010.02.074 (2010). 20206704

[b26] Annese J. *et al.* Postmortem examination of patient HM’s brain based on histological sectioning and digital 3D reconstruction. Nat. Commun. 5, doi: 10.1038/ncomms4122 (2014). PMC3916843 24473151

[b27] Duyckaerts C., Potier M.-C. & Delatour B. Alzheimer disease models and human neuropathology: similarities and differences. Acta. Neuropathol. 115, 5–38, doi: 10.1007/s00401-007-0312-8 (2008). 18038275 PMC2100431

[b28] Blanchard V. *et al.* Time sequence of maturation of dystrophic neurites associated with A beta deposits in APP/PS1 transgenic mice. Exp. Neurol. 184, 247–263, doi: 10.1016/s0014-4886(03)00252-8 (2003). 14637096

[b29] Garcia-Alloza M. *et al.* Characterization of amyloid deposition in the APPswe/PS1dE9 mouse model of Alzheimer disease. Neurobiol. Dis. 24, 516–524, doi: 10.1016/j.nbd.2006.08.017 (2006). 17029828

[b30] Chubb C. *et al.* BioVision: An application for the automated image analysis of histological sections. Neurobiol. Aging 27, 1462–1476, doi: 10.1016/j.neurobiolaging.2005.08.023 (2006). 16271803

[b31] Lebenberg J. *et al.* Validation of MRI-based 3D digital atlas registration with histological and autoradiographic volumes: An anatomofunctional transgenic mouse brain imaging study. Neuroimage 51, 1037–1046, doi: 10.1016/j.neuroimage.2010.03.014 (2010). 20226256

[b32] Bowden D. M., Song E., Kosheleva J. & Dubach M. F. NeuroNames: An Ontology for the BrainInfo Portal to Neuroscience on the Web. Neuroinformatics 10, 97–114, doi: 10.1007/s12021-011-9128-8 (2012). 21789500 PMC3247656

[b33] Graeber M. B. & Streit W. J. Microglia: biology and pathology. Acta. Neuropathol. 119, 89–105, doi: 10.1007/s00401-009-0622-0 (2010). 20012873

[b34] Dhenain M. *et al.* Characterization of *in vivo* MRI detectable thalamic amyloid plaques from APP/PS1 mice. Neurobiol. Aging 30, 41–53, doi: 10.1016/j.neurobiolaging.2007.05.018 (2009). 17588710

[b35] Petiet A. *et al.* Gadolinium-staining reveals amyloid plaques in the brain of Alzheimer’s transgenic mice. Neurobiol. Aging 33, 1533–1544, doi: 10.1016/j.neurobiolaging.2011.03.009 (2012). 21531045

[b36] Friston K. J., Frith C. D., Liddle P. F. & Frackowiak R. S. J. Comparing functional (pet) images-the assessment of significant change. J. Cereb. Blood Flow Metab. 11, 690–699, doi: 10.1038/jcbfm.1991.122 (1991). 2050758

[b37] Casteels C. *et al.* Construction and evaluation of multitracer small-animal PET probabilistic atlases for voxel-based functional mapping of the rat brain. J. Nucl. Med. 47, 1858–1866 (2006). 17079820

[b38] Nguyen P. T., Holschneider D. P., Maarek J. M. I., Yang J. & Mandelkern M. A. Statistical parametric mapping applied to an autoradiographic study of cerebral activation during treadmill walking in rats. Neuroimage 23, 252–259, doi: 10.1016/j.neuroimage.2004.05.014 (2004). 15325372 PMC4103584

[b39] Lebenberg J. *et al.* A combination of atlas-based and voxel-wise approaches to analyze metabolic changes in autoradiographic data from Alzheimer’s mice. Neuroimage 57, 1447–1457, doi: 10.1016/j.neuroimage.2011.04.059 (2011). 21571077

[b40] Boussicault L. *et al.* Impaired brain energy metabolism in the BACHD mouse model of Huntington’s disease: critical role of astrocyte-neuron interactions. J. Cereb. Blood Flow Metab. 34, 1500–1510, doi: 10.1038/jcbfm.2014.110 (2014). 24938402 PMC4158666

[b41] Schupf N. *et al.* Peripheral A beta subspecies as risk biomarkers of Alzheimer’s disease. Proc. Natl. Acad. Sci. 105, 14052–14057, doi: 10.1073/pnas.0805902105 (2008). 18779561 PMC2544577

[b42] Dauguet J. *et al.* Comparison of fiber tracts derived from *in-vivo* DTI tractography with 3D histological neural tract tracer reconstruction on a macaque brain. NeuroImage, 164, 191–204, doi: 10.1016/j.jneumeth.2007.04.017 (2007). 17604650

[b43] Dorr A. E., Lerch J. P., Spring S., Kabani N. & Henkelman R. M. High resolution three-dimensional brain atlas using an average magnetic resonance image of 40 adult C57Bl/6J mice. Neuroimage 42, 60–69, doi: 10.1016/j.neuroimage.2008.03.037 (2008). 18502665

[b44] Viola P. & Wells W. M. Alignment by maximization of mutual information. Int. J. Comput. Vis. 24, 137–154, doi: 10.1023/a:1007958904918 (1997).

[b45] Rueckert D. *et al.* Nonrigid registration using free-form deformations: Application to breast MR images. IEEE Trans. Med. Imaging 18, 712–721, doi: 10.1109/42.796284 (1999). 10534053

