# Supplementary material for: High-throughput 3D whole-brain quantitative histopathology in rodents
Source: Sci Rep. 2016 Feb 15;6:20958. doi: 10.1038/srep20958 (PMC4753455; doi:10.1038/srep20958)
Supplement: Supplementary Information [file srep20958-s3.pdf]

High-throughput 3D whole brain quantitative histopathology

Michel E. Vandenberghe, Anne-Sophie Hérard, Nicolas Souedet, Elmahdi Sadouni, Mathieu D Santin, Dominique Briet, Denis Carré, Jocelyne Schulz, Philippe Hantraye, Pierre-Etienne Chabrier, Thomas Rooney, Thomas Debeir, Véronique Blanchard, Laurent Pradier, Marc Dhenain, Thierry Delzescaux

Supplementary Information

Table of contents

1 Supplementary Figures 1

Supplementary Figure 1 1

Supplementary Figure 2 2

Supplementary Figure 3 3

Supplementary Figure 4 4

Supplementary Figure 5 5

2 Supplementary Tables 6

Supplementary Table 1 6

Supplementary Table 2 7

Supplementary Table 3 7

3 Supplementary Protocols 8

Supplementary Protocol 1 8

4 Supplmentary movies 9

Supplementary Movie 1 9

Supplementary Movie 2 9

1 SUPPLEMENTARY FIGURES

SUPPLEMENTARY FIGURE 1

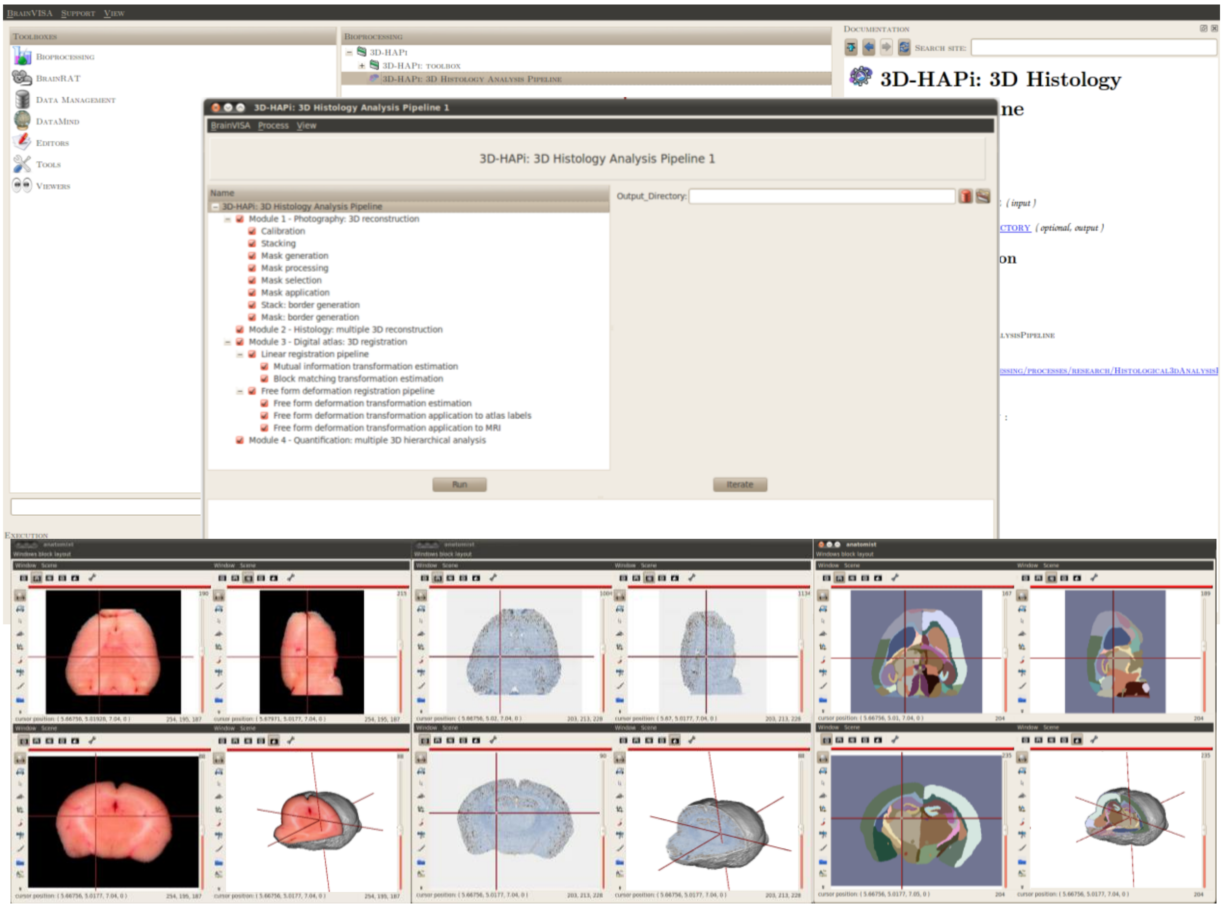

3D-HAPi pipeline. Image processing using 3D-HAPi graphical user interface (top) and multiview image visualization with Anatomist software (bottom).

SUPPLEMENTARY FIGURE 2

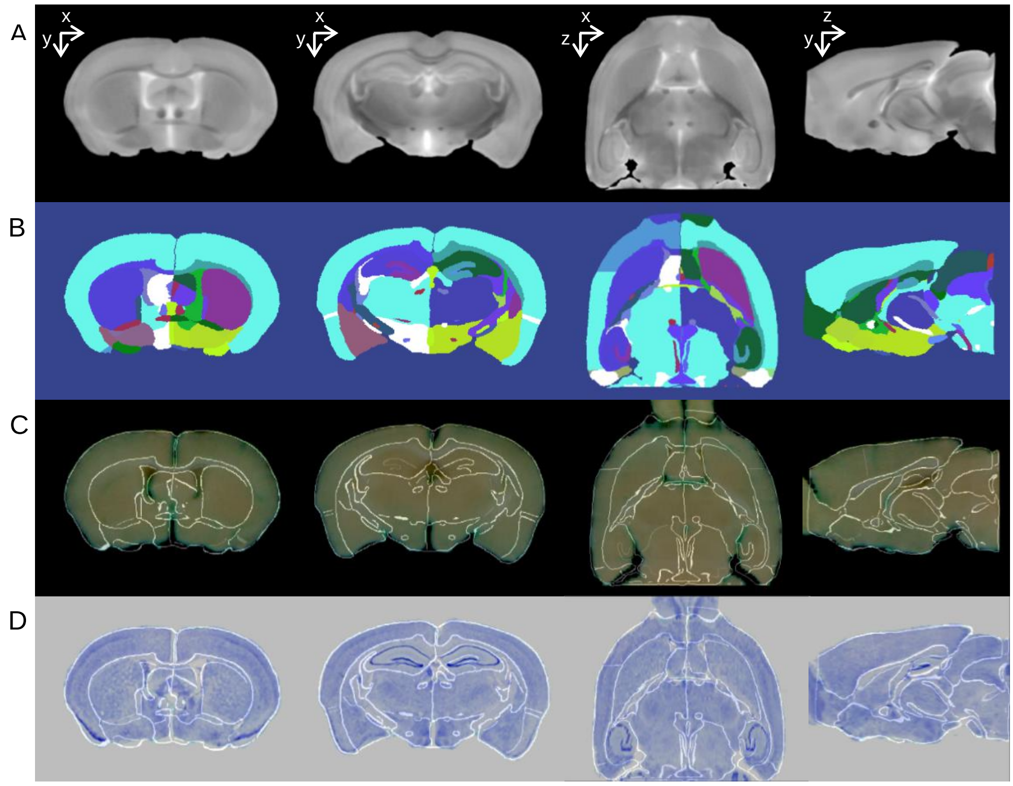

Mouse brain digital atlas registration with 3D histology. (a) Atlas MRI and (b) Atlas labels from Dorr et al., 2008. (c) Registered atlas label contours superimposed with block-face photographic volume. (d) Atlas label contours superimposed on the Nissl-stained volume.

SUPPLEMENTARY FIGURE 3

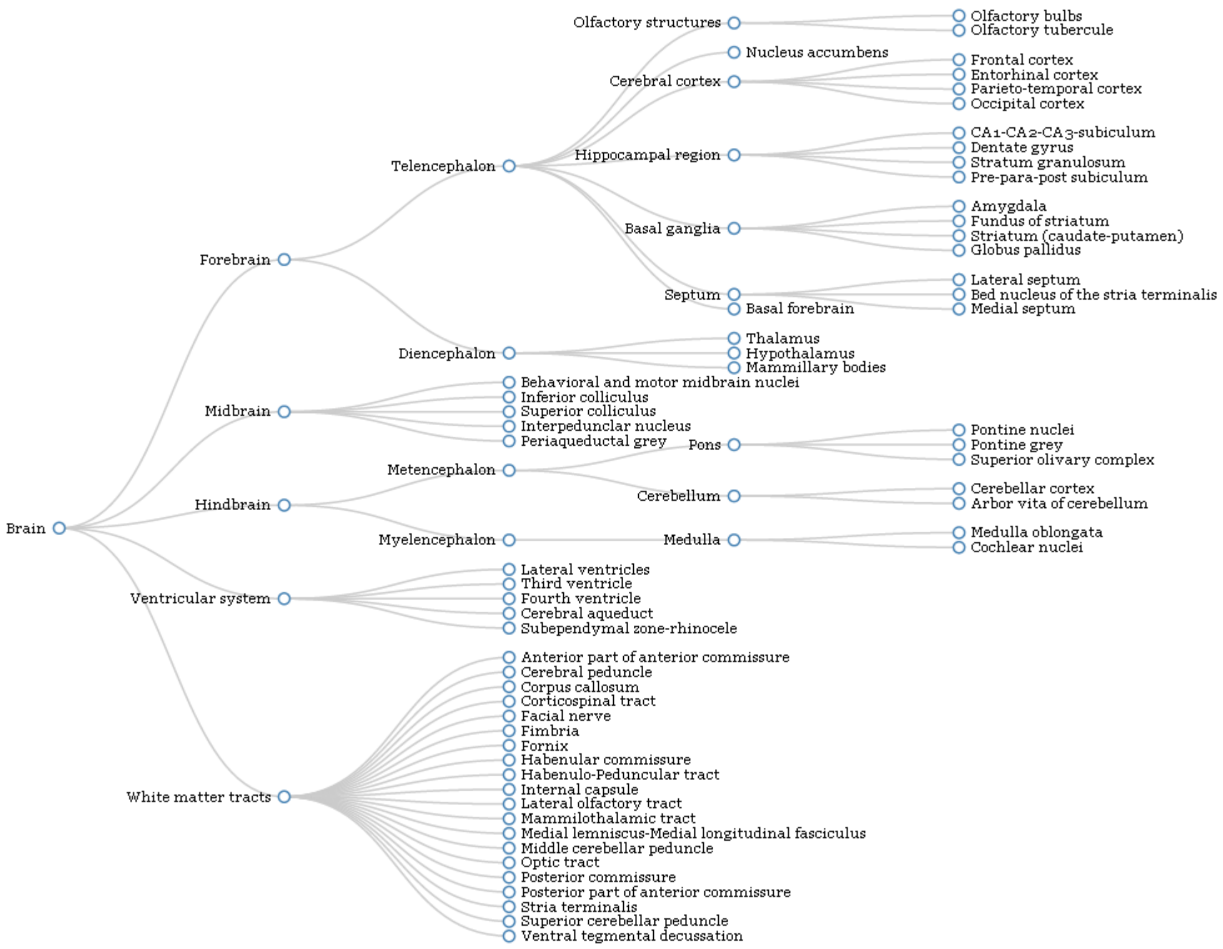

Mouse brain hierarchy. Most elementary structures correspond to the mouse brain atlas labels. Some of these structures are very small brain regions which are not necessarily relevant in studies focusing on pathological markers. We have created a brain ontology adapted from NeuroNames ontology (http://braininfo.rprc.washington.edu) so as to merge small structures into more relevant ones. This hierarchy is made available and can be downloaded along with 3D-HAPi.

SUPPLEMENTARY FIGURE 4

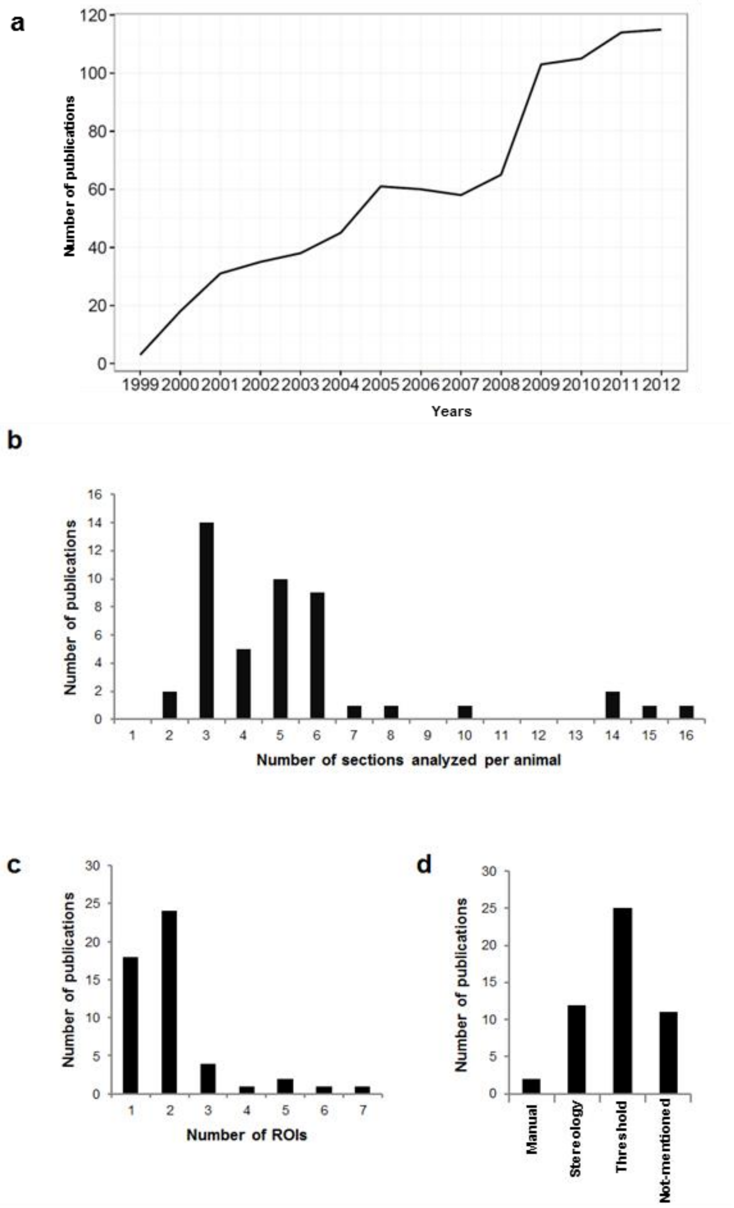

Literature survey on amyloid load quantification between 1999 and 2012. (**a**) Evolution over the last decade of the number of publications referenced on NCBI (http://www.ncbi.nlm.nih.gov/pubmed) that quantify Aβ deposition in mouse models of AD. (**b-d**) Among those publications, 51 were chosen for further analysis. Articles were selected based on journal impact factor (2012 impact factor had to be at least equal to 2) and if the quantification method was adequately described. In our publication sample, most of the scientists analyzed a total of 3 to 6 sections (**b**) and manually delineated 1 or 2 ROIs (**c**) for analysis. Once ROIs are specified, a variety of amyloid plaque segmentation methods were used (**d**). Some required heavy human interventions like manual segmentation/visual counting or stereology. Scientists mostly used image analysis software. Images were usually segmented using a threshold operation. Some publications did not specify the algorithm used by the software.

Supplementary Figure 5

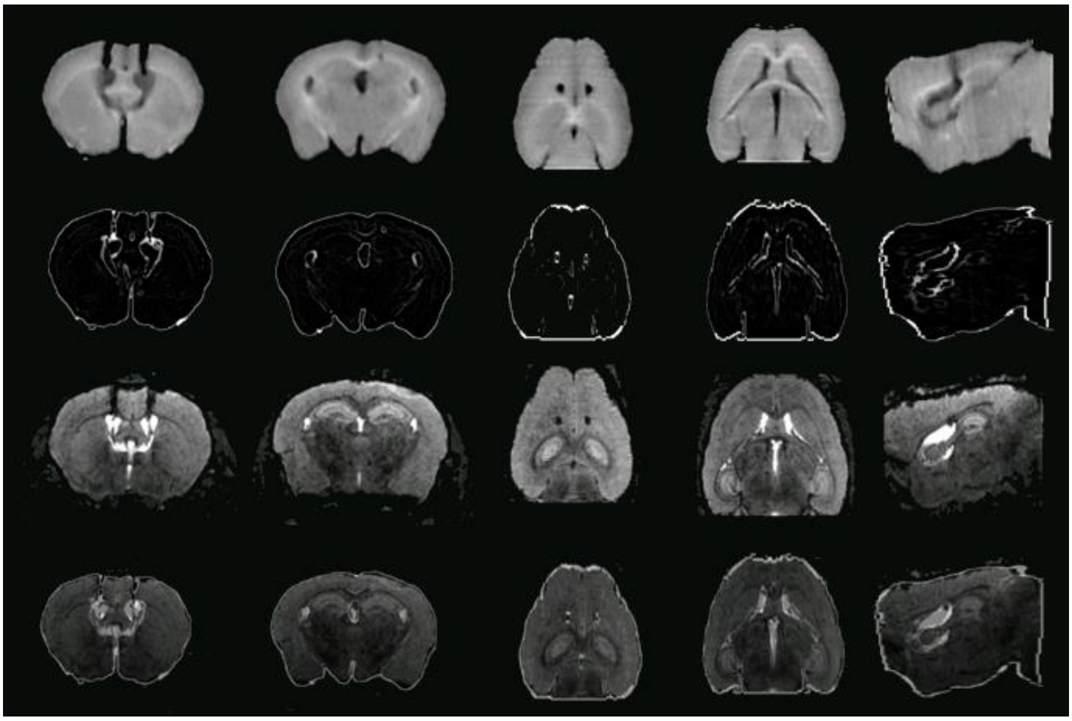

Block-face photographic volume registration with *In vivo* MRI. From top to bottom: a block-face photographic volume that have been registered in 3D with its corresponding *in vivo* MRI; contours obtained with a Deriche filter on the registered block-face photographic volume; *in vivo* MRI; superimposition of *in vivo* MRI with contours from the registered block-face photographic volume. From left to right: coronal view at the level of gadolinium injection sites; coronal view at the level of the dorsal hippocampal region; dorsal axial view at the level of gadolinium injection sites; ventral axial view; sagittal view.

SUPPLEMENTARY TABLE 1

Summarized information for the 2 datasets used in the studies.

	Dataset 1	Dataset 2		
**Transgenic mouse strains**	APP/PS1dE9	APP/PS1		PS1
**Treatment**	N.A.	13C3a<br>10 mg/kg<br>i.p. weekly injections<br>for 3 months	DM4<br>10 mg/kg<br>i.p. weekly injections<br>for 3 months	N.A.
***In vivo* imaging**	N.A.	Contrast enhanced 3D Gradient-echo MRI with intracerebroventricular injections of Gadolinium before euthanasia		
**Age at euthanasia (months)**	13.5	8		
**Number of animals**	n = 7	n = 8	n = 3	n = 4
**Block-face photography volume resolution**	27 × 27 × 80 μm^3	33 × 33 × 125 μm^3		
**Histology series**	4 series of 20-µm-thick sections:<br>- Series 1: Nissl staining<br>- Series 2: BAM10 IHC<br>- Remaining series: kept for future analysis	5 series of 25-µm-thick sections:<br>- Series 1: Nissl staining<br>- Series 2: 6E10 IHC<br>- Series 3: anti-CD68 IHC<br>- Series 4: anti-IgG IHC<br>- Series 5: anti-Iba1 IHC		
**Histology Resolution**	- Nissl series: 21 µm<br>- BAM10 series: 5 µm	- Nissl series: 21 µm<br>- 6E10 series: 5 µm<br>- A subset of 8 6E10 sections per animal: 0.35 µm<br>- Nissl, 6E10, anti-CD68, anti-Iba1 series for one APP/PS1-DM4 mouse: 0.44 µm.		
**Applications**	Mouse model characterization	13C3a immunotherapy evaluation<br>Comparison with 2D histology analysis<br>Multimodal exploration of the brain<br>*In vivo* - *ex vivo* registration		

SUPPLEMENTARY TABLE 2

F1 scores for biomarker segmentation in APP/PS1 mice.

Biomarker	Aβ peptide deposits (6E10 IHC)	Phagocytic cells (CD68 IHC)	Microglial cells (Iba-1 IHC)	Nissl bodies (Nissl staining)
**F1 score**	0.77	0.94	0.73	0.78

The F1 score measures the performance of the automatic segmentation compared to ground-truth annotations. An F1 score greater than 0.7 was considered satisfactory.

SUPPLEMENTARY TABLE 3

Amyloid lowering effect of 13C3a immunotherapy in APP/PS1 mice (dataset 2).

ROI	APP/PS1-13C3a (n = 8)	APP/PS1-DM4 (n = 3)	PS1 mice (n = 4)
Whole brain	6.61 ± 2.75 ***** °°	9.84 ± 1.80 °°	0.04 ± 0.01
Cerebral cortex	10.43 ± 3.21 ***** °°	18.48 ± 3.45 °°	0.05 ± 0.15
Striatum	1.79 ± 0.9 ***** °°	4.72 ± 0.64 °°	0.04 ± 0.02
Hippocampal region	11.06 ± 3.23 °°	16.36 ± 3.33 °°	0.03 ± 0.08
Thalamus	7.21 ± 1.55 * °°	9.51 ± 0.54 °°	0.03 ± 0.02

Data shown: mean ± SD

*p<0.05 13C3a-treated APP/PS1 mice versus DM4-treated APP/PS1 mice (Mann-Whitney tests)

°°p<0.01 versus PS1 mice (Mann-Whitney tests)

3 SUPPLEMENTARY PROTOCOLS

SUPPLEMENTARY PROTOCOL 1

APP/PS1dE9 mouse brains tissue processing, block-face photography and histology (dataset 1). Fresh brains were snap frozen and embedded in a mixture of M1 embedding matrix (Thermo Fisher Scientific) and Fast Green (Sigma-Aldrich) before being entirely cut on a CM3050S cryostat (Leica). Four batches of serial coronal brain sections (20 µm), ranging from the brain frontal pole to the end of the caudal part of the cortex, were collected, mounted on superfrost slides and quickly dried. The first series was dedicated to Nissl staining. The fourth series was dedicated to amyloid peptide aggregate staining. The remaining series were stored at -80°C until processing. Images from the surface of the block were recorded every fourth section (before each section of the first series was cut) with a digital camera (Powershot G5 Pro, Canon) at a lateral resolution of 27 µm.

These photographs were taken at the end of the cryostat wheel crank course, hence with the brain in the same position section after section. An optic fiber-ring light was fixed onto the lens of the camera. This ensured a proper and homogeneous illumination of the sample from section to section. A laptop connected to the camera was used to remotely take photographs and store images directly onto the hard disk.

For the Nissl staining series, sections of the were stained in a solution of cresyl violet (250mg / 100ml final) prepared as follows: 1.25g of cresyl violet acetate in 500ml distilled water, filter and adjust to pH = 5 with 1N NaOH. Sections were stained with the Shandon Varistain 24-4 automate (Thermo Electron Corporation) according to the following protocol: step 01, 50 % ethanol, 1 min; step 02, 70 % ethanol, 1 min; step 03, 95 % ethanol, 1 min; step 04, 100 % ethanol, 1 min; step 05, toluene, 10 min; step 06, 100 % ethanol, 2 min; step 07, 95 % ethanol, 1 min; step 08, 70 % ethanol, 1 min; step 09, 50 % ethanol, 1 min; step 10, distilled water, 1 min; step 11, cresyl violet, 3 min, step 12, distilled water, 5 sec; step 13, 50% ethanol, 30 sec; step 14, 70% ethanol, 30 sec; step 15, 95% ethanol 40 sec; step 16, 100% ethanol, 1 min; step 17, toluene, 10 min.

To highlight Aβ peptide aggregates, after post-fixation in 4% paraformaldehyde in PBS, we performed an IHC staining with BAM10 primary monoclonal antibody (Sigma-Aldrich, 1:500 dilution), a biotinylated goat anti-mouse secondary antibody (Vector Laboratories) and staining was revealed with DAB detection kit (Ventana Medical Systems, Roche). IHC experiments were performed with the automate Discovery XT (Ventana Medical Systems, Roche). All the sections were processed identically. Sections were counter-stained with Bluing Reagent (Ventana Medical Systems, Roche).

4 SUPPLMENTARY MOVIES

SUPPLEMENTARY MOVIE 1

3D rendering of blood-brain-barrier disruptions in one APP/PS1 mouse.

SUPPLEMENTARY MOVIE 2

3D rendering of superimposed Aβ load heat map with in vivo MRI in an APP/PS1 mouse.
